# Nurse-Matrons', Sisters', and Dispensers' Certificates

**Published:** 1907-11-30

**Authors:** Edward Carnall

**Affiliations:** Medical Superintendent, Newton Abbot Isolation Hospital.


### NURSE-MATRONS', SISTERS', AND DISPENSERS' CERTIFICATES.

SIR,—Those who are responsible for the appointment of a nurse-matron or sister to our small provincial hospitals, whether cottage or isolation hospitals, should, I think, appoint only those who are able to satisfy the medical man or men connected with the institution that they understand the common garden dispensing; in fact, they should possess the Dispenser's Certificate of the Apothecaries' Hall. It is very awkward if a sister cannot read what is written on the patient's bed-card or make up a simple mixture from a stock-bottle, but expect every simple mixture-lotion to be made up at the local chemist's for each patient. Of course, in some cases it is too much trouble—a chronic failing with not a few—in others it is complete ignorance. The expense to small hospitals by always sending to a chemist for each mixture is considerable. From a stock mixture of Mistura alba I ordered one day half an ounce with Tinct. Nuc. Vom. five minims, and water to the one ounce; but was told, if I wanted the patient to have it, I must send to the chemist, as there was no time for such complex work. If the hospital is outside the town it is often very awkward, and delay is experienced in getting the mixture.

Yours truly,

EDWARD CARNALL, D.P.H., M.R.C.S., Eng.,
Medical Superintendent, Newton Abbot Isolation Hospital.

